# Remote history of VTE is associated with severe COVID‐19 in middle and older age: UK Biobank cohort study

**DOI:** 10.1111/jth.15452

**Published:** 2021-07-20

**Authors:** Jana J. Anderson, Frederick K. Ho, Claire L. Niedzwiedz, Srinivasa Vittal Katikireddi, Carlos Celis‐Morales, Stamatina Iliodromiti, Paul Welsh, Pierpaolo Pellicori, Evangelia Demou, Claire E. Hastie, Donald M. Lyall, Stuart R. Gray, John F. Forbes, Jason M. R. Gill, Daniel F. Mackay, Colin Berry, John G. F. Cleland, Naveed Sattar, Jill P. Pell

**Affiliations:** ^1^ Institute of Health and Wellbeing University of Glasgow Glasgow UK; ^2^ MRC/CSO Social and Public Health Sciences Unit Institute of Health and Wellbeing University of Glasgow Glasgow UK; ^3^ Institute of Cardiovascular and Medical Sciences University of Glasgow BHF Glasgow Cardiovascular Research Centre Glasgow UK; ^4^ Centre of Women’s Health Yvonne Carter Building Queen Mary University of London London UK; ^5^ Robertson Centre for Biostatistics Institute of Health and Wellbeing University of Glasgow Glasgow UK; ^6^ School of Medicine University of Limerick Limerick Ireland

**Keywords:** COVID‐19 severity, DVT, PE, SARS‐CoV2 infection, venous thromboembolism

## Abstract

**Background:**

Venous thromboembolism (VTE) is a common, life‐threatening complication of COVID‐19 infection. COVID‐19 risk‐prediction models include a history of VTE. However, it is unclear whether remote history (>9 years previously) of VTE also confers increased risk of COVID‐19.

**Objectives:**

To investigate possible association between VTE and COVID‐19 severity, independent of other risk factors.

**Methods:**

Cohort study of UK Biobank participants recruited between 2006 and 2010. Baseline data, including history of VTE, were linked to COVID‐19 test results, COVID‐19‐related hospital admissions, and COVID‐19 deaths. The risk of COVID‐19 hospitalization or death was compared for participants with a remote history VTE versus without. Poisson regression models were run univariately then adjusted stepwise for sociodemographic, lifestyle, and comorbid covariates.

**Results:**

After adjustment for sociodemographic and lifestyle confounders and comorbid conditions, remote history of VTE was associated with nonfatal community (RR 1.61, 95% CI 1.02–2.54, *p *= .039), nonfatal hospitalized (RR 1.52, 95% CI 1.06–2.17, *p *= .024) and severe (hospitalized or fatal) (RR 1.40, 95% CI 1.04–1.89, *p *= .025) COVID‐19. Associations with remote history of VTE were stronger among men (severe COVID‐19: RR 1.68, 95% CI 1.14–2.42, *p *= .009) than for women (severe COVID‐19: RR 1.07, 95% CI 0.66–1.74, *p *= .786).

**Conclusion:**

Our findings support inclusion of remote history of VTE in COVID‐19 risk‐prediction scores, and consideration of sex‐specific risk scores.

**Essentials**

- Venous thromboembolism (VTE) is a common complication of severe COVID‐19 infection.
- COVID‐19 prediction scores include history of VTE but it is unknown if remote history is relevant.
- UK Biobank data show an independent association between remote history of VTE and severe COVID‐19.
- Our findings support inclusion of remote history of VTE in COVID‐19 risk‐prediction scores.

## 1 INTRODUCTION

Venous thromboembolism (VTE), which includes pulmonary embolism and deep vein thrombosis, is a common and a serious complication of severe COVID‐19 infection.^1^ Despite anticoagulant thromboprophylaxis being recommended and empirically used for all hospitalized COVID‐19 patients,^2^ larger studies suggest that 7% to 8% still develop VTE, as do 46% of those admitted to intensive care units.^3^ Many randomized controlled trials are now evaluating antithrombotic agents and intensities of anticoagulation for patients hospitalized with COVID‐19.^4^ Recent publications cast uncertainty about the best course of action but potentially patients who are less sick might benefit from more intense anticoagulation but for patients with severe disease it may be too late.^5^ The potential benefits of more intense anticoagulation must be weighed against the risk of bleeding, which is also increased with COVID‐19 infection.^6^, ^7^ Following discharge from hospital, there is an elevated risk of both thrombosis and hemorrhage, so universal postdischarge anticoagulation or antiplatelet therapy needs further evaluation, too.^8^

Risk stratification for VTE complications in COVID‐19 patients is needed to improve the risk‐benefit ratio of targeted anticoagulation strategies.^9^ In the most recent QCovid predictive score (https://qcovid.org/),^10^ history of VTE was identified, and included as a risk factor for both COVID‐19 severity and VTE complications after COVID‐19 infection, but no differentiation was made between remote and recent history of VTE. It is uncertain whether a remote history of VTE is a risk factor for severe COVID‐19 infection. We used UK Biobank data, collected >9 years before the COVID‐19 pandemic, to address this question.

## 2 METHODS

### 2.1 Exposure and outcomes

UK Biobank, a general population cohort, recruited participants aged 37 to 73 years between 2006 and 2010. At enrollment, participants had physical measurements taken and provided information by completing a touch‐screen questionnaire, which included questions about medical history, including self‐report of physician diagnosis of deep vein thrombosis or pulmonary embolism,^11^ here combined as VTE. COVID‐19 test results of participants living in England and hospital admissions were obtained from Public Health England^12^ and COVID‐19 deaths from death certificates, between 16 March 2020 and 22 July 2020. COVID‐19 infection was defined as more than one COVID‐19‐positive polymerase chain reaction test or COVID‐19 stated on death certificate and categorized into: nonfatal community, nonfatal hospitalized, and died from COVID‐19 (in hospital or in community). Severe COVID‐19 was defined as hospitalization for or death from COVID‐19 and ascertained by combining the latter two categories. These data were linked to the UK Biobank participants’ data collected at enrollment.

### 2.2 Covariates

Sociodemographic factors included age (continuous), sex (male/female), Townsend deprivation index (continuous), ethnicity (White, mixed, South Asian, Black, Chinese, any other). Lifestyle factors included body mass index (BMI) calculated from weight/height^2^ and categorized into underweight <18.5 kg/m^2^, normal weight 18.5 to 24.9 kg/m^2^, overweight 25.0 to 29.9 kg/m^2^, and obese ≥30.0 kg/m^2^; smoking (never, past. or current smoker); physical activity, derived from the questions about moderate and vigorous physical activity in the International Physical Activity Questionnaire, converted into metabolic equivalents (METs) and dichotomized into inactive (<600 MET min/week) and active (>600 MET min/week)^13^; and frequency of alcohol consumption (never, special occasions only, one to three times a month, once or twice a week, three or four times a week, and daily or almost daily). Self‐report of physician diagnoses included cardiovascular disease (myocardial infarction, heart failure, angina, stroke, transient ischemic attack, atrial fibrillation/flutter, valve disease) and diabetes mellitus (type 1 and type 2). Women were categorized as currently receiving exogenous estrogens (combined oral contraceptive pill or hormone replacement therapy) or not. Medication included any antiplatelet and anticoagulant medication taken regularly at the time of recruitment.

### 2.3 Statistical analyses

The characteristics of the study population, broken down by history of VTE or not, were summarized using the median and interquartile range for continuous variables and frequencies and proportions for categorical variables. Poisson regression models were run univariately, then adjusted for sociodemographic factors only (sex, age, deprivation, ethnicity), then also lifestyle factors (BMI, smoking, physical activity, and alcohol consumption frequency), and then also prevalent cardiovascular disease and diabetes at baseline. For women, use of exogenous estrogens (combined oral contraceptive pill/hormone replacement therapy or neither) at baseline was an additional covariate in the final adjusted model. Subgroup analyses were performed by sex and by antiplatelet and anticoagulant medication status at baseline. Participants with missing data on history of VTE were excluded from this study and participants with missing data for any covariates (3.3%) were excluded from the statistical analyses. Analyses were performed using STATA 14 (Stata).

## 3 RESULTS AND DISCUSSION

After excluding participants who withdrew from UK Biobank (*N* = 120), those who were recruited in assessment centers outside of England (*N* = 56 664), those who died before March 2020 when COVID‐19 testing commenced (*N* = 26 242), and those with missing information on history of VTE (*N* = 107 320), 312 378 participants were eligible for inclusion, of whom 10 034 (3.2%) reported a history of VTE at baseline. Overall, 1402 (0.45%) participants were recorded as being infected with COVID‐19 over the study period: 421 (0.14%) were nonfatal community cases, 646 (0.21%) were nonfatal hospitalized cases, and 335 (0.11%) died from COVID‐19. Therefore, 981 (0.31%) developed severe COVID‐19 defined as requiring hospitalization or death.

Participants who reported a history of VTE at baseline were older, more deprived, and more likely to be female and white (Table 1). They were less physically active, more likely to be obese and current or past smokers, more likely to have comorbid diabetes and cardiovascular disease and be on regular antiplatelet or anticoagulant therapy, but were less likely to consume alcohol weekly or daily (Table 1). Women with a history of VTE were less likely to be on exogenous estrogens at recruitment. Those with history of VTE at baseline were more likely to have subsequently had COVID‐19 (0.8% vs 0.4%) and more likely to have had severe COVID‐19 (0.5% vs 0.3%).

**TABLE 1:** Characteristics of study participants by history of venous thromboembolism

	History of VTE		*p* Value
	No *N* = 302 344	Yes *N* = 10 034	
	Median (IQR)	Median (IQR)	
Age (years)	59 (51, 64)	61 (54, 65)	<.001
Deprivation index	−2.1 (−3.6, 0.6)	−1.9 (−3.5, 1.0)	<.001
	*N* (%)	*N* (%)	
Sex			
Male	135 520 (44.8)	3899 (38.9)	<.001
Female	166 824 (55.2)	6135 (61.1)	
Ethnic group			
White	283 180 (94.1)	9570 (95.4)	<.001
South Asian	6644 (2.2)	128 (1.3)	
Black	5427 (1.8)	171 (1.7)	
Chinese	858 (0.29)	5 (0.1)	
Mixed	1844 (0.6)	59 (0.6)	
Any other	2900 (0.96)	70 (0.7)	
Smoking status			
Current	29 070 (9.7)	1089 (10.9)	<.001
Past	108 441 (36.1)	3717 (37.2)	
Never	163 213 (54.3)	5175 (51.8)	
Body mass index			
Underweight	1260 (0.4)	22 (0.2)	<.001
Normal weight	88 037 (29.7)	2113 (21.9)	
Overweight	127 918 (43.1)	3900 (40.4)	
Obese	79 518 (26.8)	3615 (37.5)	
Alcohol frequency			
Never	25 601 (8.5)	1171 (11.7)	<.001
Special occasions only	36 556 (12.1)	1508 (15.1)	
1–3 times/month	34 119 (11.3)	1182 (11.8)	
1–2 times/week	75 930 (25.2)	2377 (23.7)	
3–4 times/week	67 961 (22.5)	1968 (19.6)	
Daily or almost daily	61 465 (20.4)	1811 (18.1)	
Physically active	159 794 (52.9)	5066 (50.5)	<.001
Cardiovascular disease	20 897 (6.9)	1236 (12.3)	<.001
Diabetes	19 228 (6.4)	789 (7.9)	<.001
Anticoagulants			
Yes	2341 (0.9)	1162 (13.7)	<.001
No	247 015 (99.1)	7313 (86.3)	
Antiplatelets			
Yes	48 919 (19.6)	2261 (26.7)	<.001
No	200 437 (80.4)	6214 (73.3)	
COCP/HRT			
Yes	16 950 (10.2)	414 (6.8)	<.001
No	148 848 (89.8)	5668 (93.2)	

Note

Categorical variables compared by χ^2^ test; continuous variables compared by Mann‐Whitney *U* test.

Abbreviations: COCP, combined oral contraceptive pill; HRT, hormone replacement therapy; IQR, interquartile range; VTE, venous thromboembolism.

Univariately, history of VTE was associated with nonfatal community (RR 1.59, 95% CI 1.02–2.46, *p *= .039), nonfatal hospitalized (RR 1.94, 95% CI 1.40–2.68, *p *< .001), and severe (RR 1.76, 95% CI 1.34–2.31, *p *< .001) COVID‐19. The association with death from COVID‐19 was not significant (RR 1.42, 95% CI 0.84–2.38, *p *= .187). After adjustment for sociodemographic and lifestyle confounders as well as comorbid cardiovascular disease, the associations remained for nonfatal community (RR 1.61, 95% CI 1.02–2.54, *p *= .039), nonfatal hospitalized (RR 1.52, 95% CI 1.06–2.17, *p *= 0.024), and severe (RR 1.40, 95% CI 1.04–1.89, *p *= .025) COVID‐19 (Table 2). There were significant interactions with sex (nonfatal hospitalized COVID‐19 *p *= .009; severe COVID‐19 *p *= .010). In subgroup analysis, the associations were stronger for men and not statistically significant for women (Table 2).

**TABLE 2:** Associations between remote history of venous thromboembolism and COVID‐19 infection outcomes (*N* = 312 378)

COVID‐19 Severity	Model 1 RR (95% CI)	*p* Value	Model 2 RR (95% CI)	*p* Value	Model 3 RR (95% CI)	*p* Value	Model 3 Men only RR (95% CI)	*p* Value	Model 3 Women Only RR (95% CI)	*p* Value
Nonfatal community	1.74 (1.12–2.69)	.014	1.63 (1.04–2.57)	.034	1.61 (1.02–2.54)	.039	2.23 (1.18–4.23)	.014	1.28 (0.67–2.44)	.456
Hospitalized nonfatal	1.91 (1.37–2.64)	<.001	1.55 (1.08–2.22)	.018	1.52 (1.06–2.17)	.024	1.88 (1.18–2.98)	.007	1.07 (0.59–1.96)	.827
Died from COVID‐19	1.24 (0.74–2.08)	.415	1.22 (0.73–2.04)	.456	1.21 (0.72–2.03)	.467	1.32 (0.68–2.57)	.405	1.06 (0.46–2.41)	.893
Severe (hospitalized/died from COVID‐19)	1.66 (1.26–2.18)	<.001	1.42 (1.06–1.91)	.019	1.40 (1.04–1.89)	.025	1.68 (1.14–2.42)	.009	1.07 (0.66–1.74)	.786

Model 1: Adjusted for sociodemographic factors at baseline (sex, age, deprivation, ethnicity).

Model 2: Also adjusted for lifestyle factors at baseline (body mass index, smoking, physical activity, alcohol consumption).

Model 3: Also adjusted for comorbid cardiovascular disease (myocardial infarction, heart failure, angina, stroke, transient ischemic attack, atrial fibrillation/flutter, valve disease) and prevalent diabetes mellitus; also adjusted for use of exogenous oestrogens in women only.

Participants with a remote history of VTE not taking antithrombotic therapy at baseline had a higher risk of nonfatal hospitalized (RR 2.05, 95% CI 1.29–3.24, *p *= .002) and severe COVID‐19 (RR 1.84, 95% CI 1.25–2.70, *p *= .002), with similar but nonsignificant trends for deaths from (RR 1.46, 95% CI 0.72–2.96, *p *= .292) or nonfatal community COVID‐19 (RR 1.74, 95% CI 0.95–3.20, *p *= .075). These associations were absent for those with a remote history of VTE and on antiplatelet and/or anticoagulant medication at baseline: nonfatal hospitalized (RR 1.31, 95% CI 0.71–2.43, *p *= .386) and severe COVID‐19 (RR 1.25, 95% CI 0.77–2.01, *p *= .369), deaths from (RR 1.15, 95% CI 0.53–2.48, *p *= .729), or nonfatal community (RR 0.85, 95% CI 0.30–2.40, *p *= .764) COVID‐19.

Remote history of VTE was associated with hospitalized and severe COVID‐19 disease, independent of sociodemographic characteristics, lifestyle, and comorbidity. The excess risk of hospitalization with COVID‐19 appeared somewhat higher, although the confidence intervals overlapped. This might reflect incorrect attribution of the cause of deaths by clinicians for people who died in the community; either the relationship to COVID‐19 was missed due to lack of testing or deaths from respiratory disease were wrongly ascribed to COVID‐19. Interaction tests and subgroup analyses show that the association between remote history of VTE and COVID‐19 hospitalizations was specific to men; with no association in women irrespective of their use of exogenous estrogens. Clinical guidelines recommend that hormone therapy should be avoided in women with a history of VTE.^14^ Men with a remote history of VTE were more likely to have a positive test for COVID‐19 and to be hospitalized or die from it. Women and men are presumably at similar risk of getting COVID‐19 infection, but men are known to have a higher risk of developing severe disease. Male sex may amplify the prothrombotic effects of COVID‐19 infection and consequently of severe disease.

Also, patients with a remote history of VTE treated with antiplatelet agents or anticoagulants appeared to be protected from developing more severe COVID‐19. This is interesting, because activation of hemostatic pathways, in the micro‐ or macro‐circulation, may be an important mechanism determining the severity of COVID‐19. Antithrombotic prophylaxis with, for instance, a low dose of a novel oral anticoagulant and/or aspirin might reduce the risks of developing severe COVID‐19.^15^ In the COMPASS trial, patients with stable atherosclerotic vascular disease were randomized to aspirin 100 mg/day alone, aspirin 100 mg/day plus rivaroxaban 2.5 mg twice daily, or rivaroxaban 5 mg twice daily. The trial was stopped early because of benefit, including reductions in stroke, venous thromboembolism, cardiovascular, and all‐cause mortality among patients assigned combination therapy compared with aspirin alone.^15^ If the sequence of events leading to severe COVID‐19 is indeed infection, systemic inflammation, endothelial damage, and finally microvascular thrombosis and occlusion in the pulmonary, renal, cardiac, and other circulations, then intervention before the development of this final step could prevent the most serious consequences of COVID‐19. To date, trials of antithrombotic agents in late stages of the disease with widespread activation of hemostasis have not improved COVID‐19 outcomes (RECOVERY trial; www.recoverytrial.net). This does not preclude the possibility that much earlier intervention might prevent progression to more severe disease.

The study has several strengths. To our knowledge, this is the first study to investigate whether a remote (>9 years previously) history of VTE is associated with severe COVID‐19 disease. UK Biobank is a large, well‐characterized general population cohort (10 074 participants had a remote history of VTE) and we were able to adjust for a wide range of potential confounders and were powered to undertake subgroup analyses.

There are several limitations to our study. UK Biobank is not representative of the general population in terms of sociodemographic and lifestyle factors; however, estimates of effect size are nonetheless generalizable.^16^ History of VTE and covariates were measured at baseline and may have changed over time; however, we have focused on remote (>9 years previously) history of VTE. We had no data on VTE events or use of antithrombotic/anticoagulant medication at the time of infection. In addition, a large number of patients was excluded because of missing information on history of VTE. Such individuals were younger, from more deprived areas, more likely to be non‐White, to drink alcohol, but also be more active and have lower BMI. It is possible these differences could have biased our results.

Finally, ascertainment of COVID‐19 was dependent on the testing strategy in operation at the time. In our report, 70% of cases were severe, which is a much higher proportion than expected. It is likely that participants with mild or asymptomatic infections did not seek medical attention and were therefore not tested and, consequently, were included in the comparison group. This detection bias might have diluted the association between remote history of VTE and COVID‐19 infection. However, it is unlikely that VTE would predispose to being infected with COVID‐19. It is far more likely that having a remote history of VTE identifies people who, when infected, develop more severe manifestations of the disease.^12^ Also, people with remote history of VTE were more likely also to have other cardiovascular problems. It is possible that the threshold for admission was lower in these patients, which might have strengthened the relationship between remote history of VTE and more severe COVID‐19.

## 4 CONCLUSIONS

Our findings support inclusion of any history of VTE, including remote, in the QCovid risk score and other risk scores. They show that the risk was specific to men. The lack of an association in women needs to be corroborated in other studies but sex‐specific risk scores should be considered. These findings also support testing anticoagulants and antiplatelet agents in the prevention of more severe COVID‐19 outcomes. Individuals with a history of VTE should be considered a potential priority group for vaccination.

## CONFLICT OF INTEREST

There is no conflict of interest to report.

## AUTHOR CONTRIBUTIONS

Jill P. Pell, Naveed Sattar, and John G.F. Cleland formalized the original concept. Jana J. Anderson undertook the statistical analyses. All authors interpreted the results. Jana J. Anderson, Jill P. Pell, and Naveed Sattar drafted the manuscript. All authors commented on draft manuscript, revised the final manuscript, and approved the final version for submission.

## ETHICAL APPROVAL

UK Biobank received ethical approval from the North West Multi‐centre Research Ethics Committee (REC 11/NW/03820).

## References

[1] Pellicori P , Doolub G , Wong CM , et al. COVID‐19 and its cardiovascular effects: a systematic review of prevalence studies. Cochrane Database Syst Rev. 2021;3:CD013879. 10.1002/14651858.CD013879. PMID: 33704775. 33704775 PMC8078349

[2] Spyropoulos AC , Levy JH , Ageno W , et al. Scientific and Standardization Committee communication: clinical guidance on the diagnosis, prevention, and treatment of venous thromboembolism in hospitalized patients with COVID‐19. J Thromb Haemost. 2020;18(8):1859‐1865. 10.1111/jth.14929 32459046 PMC7283841

[3] Fontana P , Casini A , Robert‐Ebadi H , Glauser F , Righini M , Blondon M . Venous thromboembolism in COVID‐19: systematic review of reported risks and current guidelines. Swiss Med Wkly. 2020;21(150):w20301. 10.4414/smw.2020.20301. PMID: 32640479. 32640479

[4] Talasaz AH , Sadeghipour P , Kakavand H , et al. Recent randomized trials of antithrombotic therapy for patients with COVID‐19: JACC state‐of‐the‐art review. J Am Coll Cardiol. 2021;77(15):1903‐1921. 10.1016/j.jacc.2021.02.035. Epub 2021 Mar 16. PMID: 33741176; PMCID: PMC7963001. 33741176 PMC7963001

[5] Berger JS , Connors JM . Anticoagulation in COVID‐19: reaction to the ACTION trial. Lancet. 2021;397(10291):2226‐2228. 10.1016/S0140-6736(21)01291-5. PMID: 34119049; PMCID: PMC8192086. 34119049 PMC8192086

[6] Al‐Samkari H , Karp Leaf RS , Dzik WH , et al. COVID‐19 and coagulation: bleeding and thrombotic manifestations of SARS‐CoV‐2 infection. Blood. 2020;136(4):489‐500. 10.1182/blood.2020006520. PMID: 32492712; PMCID: PMC7378457. 32492712 PMC7378457

[7] Halaby R , Cuker A , Yui J , et al. Bleeding risk by intensity of anticoagulation in critically ill patients with COVID‐19: a retrospective cohort study. J Thromb Haemost. 2021;19(6):1533‐1545. 10.1111/jth.15310 33774903 PMC8250316

[8] Patell R , Bogue T , Koshy A , et al. Postdischarge thrombosis and hemorrhage in patients with COVID‐19. Blood. 2020;136(11):1342‐1346. 10.1182/blood.2020007938 32766883 PMC7483433

[9] Hunt BJ , De Paula EV , McLintock C , Dumantepe M . Prophylactic anticoagulation for patients in hospital with covid‐19. BMJ. 2021;372:n487. 10.1136/bmj.n487 33608304

[10] Clift AK , Coupland CAC , Keogh RH , et al. Living risk prediction algorithm (QCOVID) for risk of hospital admission and mortality from coronavirus 19 in adults: national derivation and validation cohort study. BMJ. 2020;20(371):m3731. 10.1136/bmj.m3731. PMID: 33082154; PMCID: PMC7574532. PMC7574532 33082154

[11] Sudlow C , Gallacher J , Allen N , et al. UK biobank: an open access resource for identifying the causes of a wide range of complex diseases of middle and old age. PLoS Med. 2015;12(3):e1001779. 25826379 10.1371/journal.pmed.1001779 PMC4380465

[12] Armstrong J , Rudkin J , Allen N , et al. Dynamic linkage of COVID‐19 test results between Public Health England’s Second Generation Surveillance System and UK Biobank. Microbial Genomics. 2020;6(7):mgen000397. 10.1099/mgen.0.000397 PMC7478634 32553051

[13] World Health Organisation . Global recommendations on physical activity for health. Available at: http://apps.who.int/iris/bitstream/10665/44399/1/9789241599979_eng.pdf. (Accessed: 14 February 2021)

[14] Gialeraki A , Valsami S , Pittaras T , Panayiotakopoulos G , Politou M . Oral contraceptives and HRT risk of thrombosis. Clin Appl Thromb Hemost. 2018;24(2):217‐225. 10.1177/1076029616683802. Epub 2017 Jan 4. PMID: 28049361; PMCID: PMC6714678. 28049361 PMC6714678

[15] Eikelboom JW , Connolly SJ , Bosch J , et al. Rivaroxaban with or without aspirin in stable cardiovascular disease. N Engl J Med. 2017;377(14):1319‐1330. 10.1056/NEJMoa1709118. Epub 2017 Aug 27. PMID: 28844192. 28844192

[16] Fry A , Littlejohns TJ , Sudlow C , et al. Comparison of sociodemographic and health‐related characteristics of UK biobank participants with those of the general population. Am J Epidemiol. 2017;186(9):1026‐1034. 10.1093/aje/kwx246. PMID: 28641372; PMCID: PMC5860371. 28641372 PMC5860371

